# Effects of High Glucose Concentrations on PC12 Cells: Possible Implications on Neurodegeneration

**DOI:** 10.3390/cimb47100801

**Published:** 2025-09-26

**Authors:** Claudia Cannas, Grazia Galleri, Laura Doro, Ilaria Campesi, Alessandra Tiziana Peana, Rossana Migheli

**Affiliations:** 1 Department of Medicine, Surgery and Pharmacy, University of Sassari, Viale San Pietro 43/b, 07100 Sassari, Italy; c.cannas1@studenti.uniss.it (C.C.); l.doro@studenti.uniss.it (L.D.); apeana@uniss.it (A.T.P.); 2 Department of Biomedical Sciences, University of Sassari, Viale San Pietro 43/b, 07100 Sassari, Italy; galleri@uniss.it (G.G.); icampesi@uniss.it (I.C.)

**Keywords:** glucose, neurodegeneration, PC12 cells, oxidative stress, apoptosis

## Abstract

Hyperglycemia, which arises in type 1 or 2 diabetes, leads to different complications, such as macrovascular disease, nephropathy, retinopathy, and neuropathy. In addition, different cognitive variations are associated with type 1 diabetes. Long-term changes in glucose metabolism might induce effects on the central nervous system (CNS) such as reduced mental performance and loss of consciousness, which could be implicated in neurotoxicity. The direct impact of hyperglycemia and elevated glucose concentrations on neuronal cells remains to be fully elucidated, primarily due to the multifaceted mechanisms underlying glucose neurotoxicity, including apoptosis, oxidative stress, and alterations in signaling cascades. The multifaceted mechanisms further complicate the study of the relationship between diabetes and neurodegeneration. Research in this field is continually advancing, with the aim of investigating these eventual connections and developing more effective preventive and therapeutic strategies. The present study aims to assess the damage induced by different glucose concentrations (from 25 to 150 mM) in a neuronal model, such as PC12 cells, rat pheochromocytoma cells. In glucose-exposed PC12 cells, we have tested oxidative stress, apoptosis, and cell migration by (a) viability screening, (b) intracellular levels of anion superoxide (O_2_^−^), (c) extracellular levels of MDA and nitrites, (d) apoptosis, and (e) the wound healing assay. By the cell viability assay, it has emerged that glucose (25–150 mM) showed a stronger effect at the highest concentrations (100 and 150 mM). The increase in MDA and O_2_^−^ levels was determined in PC12 cells treated with high glucose concentrations (6.5–8.8 fold for MDA). High concentrations (100 and 150 mM) significantly reduced the expression of full-length caspase-3 (2.8-fold and 4.2-fold decrease at 24 and 72 h) and caspase-9 (3.4-fold and 2.8-fold decrease at 24 h and 5-fold decrease at 72 h) compared with control conditions. Finally, the wound healing assay showed different scenarios during the several time points. Indeed, the wound closure rate was reduced in a dose-dependent manner (24 h: control 18%, G 50 mM 9%, 100 and 150 mM 8%; 48 h: control 26%, G 50 mM 20%, G 100 mM 13%, 150 mM 11%), following the treatment with three concentrations considered (50, 100, 150 mM). The results obtained in these experimental conditions highlight that glucose, at high concentrations, induced cell damage and corroborate the hypothesis that it could be involved in neurodegenerative diseases.

## 1. Introduction

Glucose and its homeostasis are of predominant importance in the proper functioning and the energy requirements of vital organs are met [1]. This fundamental and continuously supplied molecule contribute to the production of ATP, neurotransmitter synthesis such as serotonin, dopamine, and norepinephrine, regulation of neuronal function, and oxidative stress [2]. Among the organs in mammalians, the brain needs glucose as its main source of energy. It represents the main user of glucose, consuming around 20% of glucose-derived energy [2,3]. Indeed, according to the selfish brain theory, the brain energy supply has priority in the competition between organs within human body for the availability of energy sources [4]. This organ is able to control its own energy supply, competing with other organs, affecting the peripheral metabolism and food intake according to its needs [5]. Abnormalities in glucose metabolism, particularly in the context of hyperglycemia, can lead to several systemic complications, including hypertension, dyslipidemia, kidney dysfunction, and neuropathy [6,7,8,9]. Hyperglycemia, typically occurs in both type 2 diabetes (T2D), characterized by insulin resistance [10,11], and type 1 diabetes (T1D), which results from autoimmune destruction of pancreatic β cells responsible for insulin production [12]. Notably, insulin resistance in T2D and insulin deficiency in T1D are both associated with cognitive impairments, affecting functions such as learning, memory, mental flexibility, and attention [6,13,14,15]. It has been reported in the literature that abnormalities in blood glucose levels, both hyperglycemia and hypoglycemia, could affect cognitive functions and brain structure [16]. Chronic hyperglycemia has been shown to lead to a fourfold increase in neuronal glucose levels. Intracellular glucose metabolism can cause neuronal damage; a phenomenon often referred to as glucose neurotoxicity [17]. Glucose can cross the blood–brain barrier through transporter proteins, for this reason, extracellular glucose levels influence its neuronal uptake [18]. The presence of glucose transporters (GLUTs) at the blood–brain barrier is of particular significance, as it ensures the continuous high glucose and energy demands of the brain are met [19].

As demonstrated in Allen et al. [18] several molecular mechanisms are involved in glucose neurotoxicity, including apoptosis and oxidative and nitrosative stress, combining free radical generation and impaired free-radical scavenging, which glucose promote. The alteration in signaling cascades, such as the activation of mitogen-activated protein kinases through free radicals and reactive carbonyls of the oxidation of glucose, and the abnormal glycation of proteins and other macromolecules, which forms advanced glycation products (AGEs-advanced glycation end products), are also involved in glucose neurotoxicity [17]. Consequently, according to several authors, elevated neuronal glucose levels have been shown to be neurotoxic, foster a neurodegenerative environment and leading to neuronal dysfunction, progressive decline in neuronal networks, and cognitive impairment [6]. This study investigated the effects of high glucose concentrations on PC12 cells, a widely used neuronal cell model, derived from rat pheochromocytoma [20,21]. The aim was to determine whether glucose at high concentrations contributed to cellular damage. To assess glucose-induced PC12 injury, we evaluated (I) cell viability, (II) apoptosis through the analysis of full-length caspase-3 and -9 expression, (III) lipid peroxidation (Malondialdehyde (MDA) levels), (IV) nitrite levels, (V) reactive oxygen species (ROS) and superoxide anion (O_2_^−^) production, and (VI) cell migration.

## 2. Materials and Methods

### 2.1. Cell Culture

PC12 cells are derived from a pheochromocytoma cell line (ATCCCRL-1721; passages 12–25) of the rat adrenal medulla. The cells were maintained at 37 °C in a 95% humidified atmosphere and 5% CO_2_ in 100 mm plastic culture Petri dishes in Dulbecco’s-modified eagle medium (DMEM)/F12 supplemented with 10% horse serum, 5% fetal bovine serum, and 1% of penicillin/streptomycin as reported by Rassu et al. [22].

### 2.2. Cell Viability Measurement

Some experiments were performed to determine whether glucose at different concentrations (25–150 mM) had cytotoxic effects on PC12 cells by measuring cell viability. Cell viability was assessed using the Trypan Blue exclusion method.

Prior to each experiment, a 0.4% solution of Trypan Blue (Sigma Aldrich, T-5526, Rockville, MD, USA) was prepared in 1× PBS. Cells cultured in 24-well plates were detached, and 20 μL of the Trypan Blue solution was added to 80 μL of cell suspension. The mixture was gently mixed to ensure thorough incorporation. Viable cells were counted in duplicate wells using a hemocytometer (Burker chamber), and each experiment was independently repeated three times. Cell viability was expressed as the percentage of viable cells (unstained by Trypan Blue) [23].

### 2.3. ROS and O_2_^−^ Detection

Intracellular ROS and O_2_^−^ content were evaluated using the total ROS detection kit (ENZ-51,011, ENZO, Enzo Life Sciences, Euroclone S.p.A.,20016,Pero, Milan, Italy.). PC12 cells were seeded in 6-well plates. After 24 and 72 h of treatment, the cells were mechanically detached, using their same medium. The contents of each well were harvested, and the wells were washed with 1 mL of PBS. The cell suspension was centrifuged at 1200 rpm for 10 min [24].

A total ROS/superoxide detection kit (Enzo Life Sciences, Farmingdale, NY, USA) was used to measure intracellular ROS and superoxide anion. Briefly, the cells were stained for 30 min at room temperature in the dark ROS and superoxide-sensitive fluorescent dyes and subsequently assayed by flow cytometry FACSCanto II (BD Biosciences, San Jose, CA, USA). Double staining with fluorescein isothiocyanate (ROS/FITC) and phycoerythrin (superoxide/PE) was carried out, and data were assessed by DIVA software version 6.3 (BD Biosciences, San Jose, CA, USA). All experiments were performed in triplicate.

### 2.4. Malondialdehyde (MDA) Determination

Lipid peroxidation levels were assessed as described in Campesi et al. [25]. Briefly, 50 μL of supernatant (culture media) were mixed with 50 μL of acetic acid (1:3 dilution in H_2_O), 40 μL of 10% (*w*/*v*) sodium dodecyl sulfate, and 50 μL of 50 mM Tris-HCl. After centrifugation at 13,000 rpm for 5 min at 24 °C, 150 μL of a 1% thiobarbituric acid (TBA) solution in acetic acid (diluted 1:3 in H_2_O) and 1N NaOH (1:1 ratio) were added. The samples were then boiled for 60 min, cooled on ice for 10 min, and further treated with 150 μL of acetic acid (1:3 dilution), followed by a second centrifugation at 13,000 rpm for 10 min at 4 °C. Finally, 100 μL of the supernatant was used to measure absorbance at 535 nm using a spectrophotometer. MDA concentrations were calculated from a calibration curve of MDA standards (50, 25, 10, and 5 μM). All experiments were performed in triplicate.

### 2.5. Nitrite Determination

Nitrites, which are stable end products of nitric oxide (NO) metabolism, were measured in 50 μL of supernatant (culture media) using the Griess reaction. Briefly, 50 μL of Griess reagent 1 (2% sulfanilamide in H_3_PO_4_) and Griess reagent 2 (0.2% N-(1-naphthyl)ethylenediamine dihydrochloride in Milli-Q water), in a 1:1 ratio, were added to 50 μL of the sample. After 15 min of incubation in the dark, absorbance was measured at 535 nm. Nitrite concentrations were calculated using a sodium nitrite standard curve ranging from 1 to 50 μM. All experiments were performed in triplicate.

### 2.6. Western Blotting

For Western blot analysis, 25 μg of protein from cellular lysates—quantified using the BCA assay kit (Thermo Scientific, Waltham, MA, USA)—was separated by electrophoresis on a 4–15% SDS-PAGE gel and subsequently transferred onto a PVDF membrane using the Trans-Blot Turbo system (Bio-Rad, Milan, Italy). Membranes were blocked for 2 h at room temperature in a 5% (*w*/*v*) skim milk solution prepared in TBST (150 mM NaCl, 1 M Tris, and 0.1% Tween-20, pH 7.6). Following blocking, membranes were incubated overnight at 4 °C with the following primary antibodies: mouse anti-caspase-9 (BK9508S) (Cell Signaling Technology, Milan, Italy), diluted 1:1000 in milk; rabbit anti-caspase-3 (sc-98785); and mouse anti-Hsp90 (Santa Cruz Biotechnology, Bergheim, Germany), diluted 1:500 in milk. The next day, membranes were washed three times with TBST (5 min each) and then incubated for 1 h at room temperature with the appropriate HRP-conjugated secondary antibody (Cell Signaling Technology, Milan, Italy) diluted 1:2000 in milk. Protein bands were visualized using the Bio-Rad ChemiDoc imaging system (Berkeley, CA, USA), and densitometric analysis was performed using the associated software. Protein expression levels were normalized to α-actin, used as a loading control. All experiments were performed in triplicate.

### 2.7. Wound Healing Assay

Cells were grown to confluence in 12-well plates in a complete medium. Confluent cells were manually scratched in each well using a p10 pipette tip, subsequently treated with different concentrations of glucose (50, 100, 150 mM), and cultured for 48 h. Photographs were taken just after scratching and after 6, 9, 24, 30, and 48 h of incubation at a 4× magnification. The percentage of wound closure was calculated using Image J 1.53tsoftware by measuring the wound area at each time point compared with the initial area measured at the time of the scratch. Each sample was assayed in duplicate [25].

### 2.8. Statistical Analysis

All experimental data are expressed as mean values ± SD. In the presence of overall significant main effects and interactions (*p* values < 0.05 or *p* < 0.01 or *p* < 0.001) following one-way-analysis of variance (ANOVA), Bonferroni multiple comparisons test as post hoc, was performed. All data were evaluated by using Graph-Pad Prism 10.0 software (Inc, San Diego, CA, USA). Linear regression analysis was performed by plotting time against the percentage of wound closure and comparing slope variations through a global test of coincidence using Graph-Pad Prism 10.0 software (Inc, San Diego, CA, USA).

## 3. Results

### 3.1. Effects of Glucose on Cell Viability

**Figure 1:**
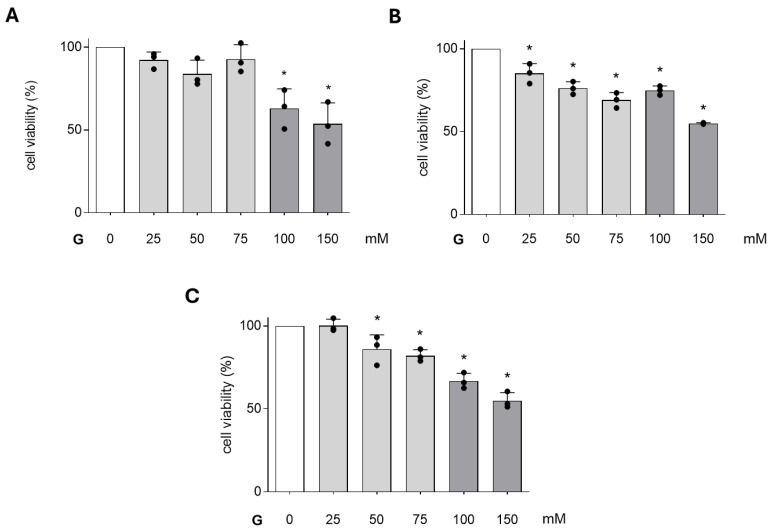
Effects of different glucose concentrations on cell viability of PC12 cells by Trypan Blue exclusion method after 24 h-exposure (**A**), 48 h-exposure (**B**), and 72 h-exposure (**C**). Data are reported as the means ± SD of at least 3 samples for each condition. * *p* < 0.05 vs. control.

Figure 1 shows the effects of different concentrations of glucose on PC12 cell viability after 24, 48, and 72 h of incubation, assessed using the Trypan Blue exclusion method. After 24 h of incubation, glucose significantly reduced cell viability at the highest concentrations (100 and 150 mM) by 38% and 47%, respectively, compared with the control (Figure 1A).

Conversely, the other concentrations exhibited no statistically significant impact. At 48 h of incubation, all the concentrations of glucose significantly reduced cell viability, demonstrating its cytotoxic effect on PC12 cells (Figure 1B). Following a 72 h incubation, a significant decline in cell viability was observed, with 50, 75, 100, and 150 mM glucose, resulting in respective reductions of 15%, 18%, 34%, and 52% when compared with the control (Figure 1C). From the obtained results, it has emerged that the glucose concentrations of 100 and 150 mM were the most significant after 24 h and 72 h of exposure, and for this reason, they were used to perform the subsequent experiments.

### 3.2. Effects of Glucose on ROS Levels

**Figure 2:**
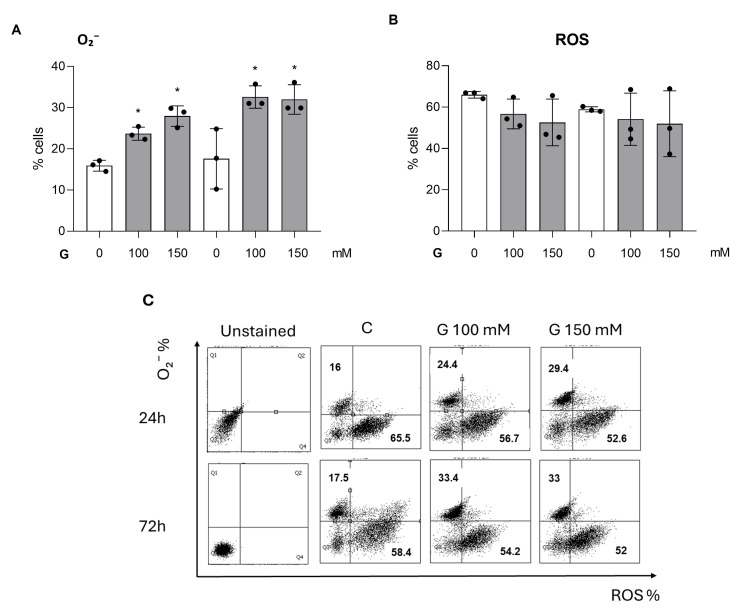
Bar graphs showing profiling of O_2_^−^ (**A**) and ROS (**B**) by flow cytometry in PC12 cell (*p* < 0.01 *). Data represent the percentage of PC12 cells positive to O_2_^−^ and ROS, following treatment with 100 mM and 150 mM glucose concentrations at 24 h and 72 h. Data are reported as the means ± SD of at least 3 samples for each condition. (**A**,**B**). Dot spot graph (**C**) shows unstained controls (from left) followed by untreated and treated samples and represents one out of three independent experiments.

Flow cytometry analysis reveals that O_2_^−^ levels increase in glucose-treated PC12 cells with respect to the control (Figure 2A,C).

Conversely, ROS does not show a significant difference between treatments and the control (Figure 2B,C).

### 3.3. Effects of Glucose on Malondialdehyde (MDA) Levels

**Figure 3:**
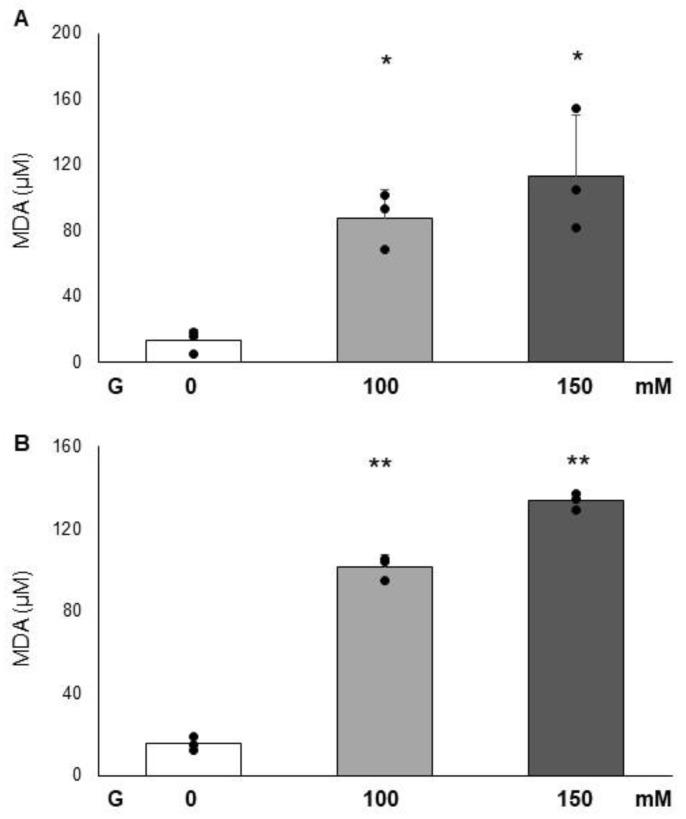
Effect of glucose (100 and 150 mM) on MDA levels after 24 h (panel (**A**)), and after 72 h (panel (**B**)) on PC12 cells. Data are reported as the means ± SD of at least 3 samples for each condition. * Represent a *p* < 0.05, and ** *p* < 0.001 versus control PC12 cells; represents a *p* < 0.05 versus G100 mM.

MDA detection was used to evaluate the contribution of glucose at the highest concentrations (100 and 150 mM) to lipid peroxidation (Figure 3) at two time points: 24 and 72 h. MDA levels in the supernatant of cells incubated with glucose (100 and 150 mM) differed significantly from those of the control after both 24 and 72 h of exposure. At the first time point (24 h exposure), both glucose concentrations caused a significant increase in MDA levels (*p* < 0.05), with 6.8-fold and 8.8-fold increases for 100 mM and 150 mM glucose, respectively (Figure 3A). Similarly, after 72 h of exposure, both concentrations induced a significant rise in MDA levels (*p* < 0.05), with 6.5-fold and 8.6-fold increases for 100 mM and 150 mM glucose, respectively (Figure 3B). Moreover, a statistically significant difference was observed between 100 mM and 150 mM glucose at 72 h (Figure 3B).

### 3.4. Effects of Glucose on Nitrite Levels

**Figure 4:**
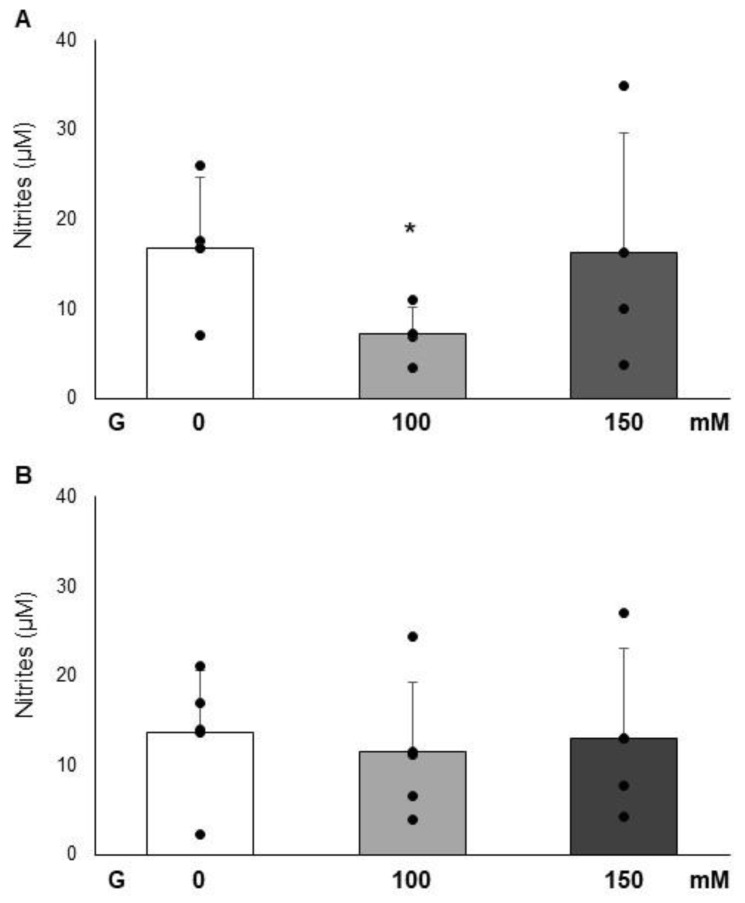
Effect of glucose (100 and 150 mM) on nitrite levels after 24 h (panel (**A**)), and after 72 h (panel (**B**)) on PC12 cells. Data are reported as the means ± SD of at least 3 samples for each condition. * Represent a *p* < 0.05 versus control PC12 cells.

As shown in Figure 4, nitrite levels in the supernatant of cells incubated with glucose (100 and 150 mM) significantly differ with respect to those in the control only after 24 h of incubation with glucose 100 mM. In the first experimental condition (24 h time exposure), glucose of 100 mM induces a significant decrease of -2.4 fold (*p <* 0.05) in nitrite levels (Figure 4A). At the second time point, (72 h time exposure) all the experimental conditions did not significantly differ from each other (Figure 4B).

### 3.5. Effects of Glucose on Full-Length Caspases-3 and -9

**Figure 5:**
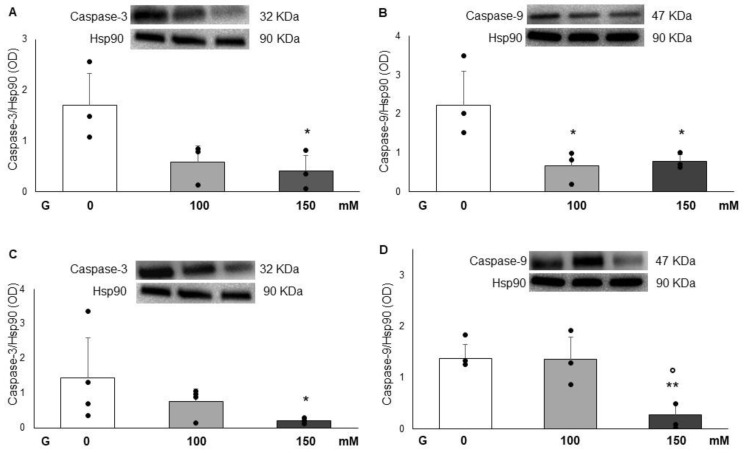
Effect of glucose (100 and 150 mM) on full-length caspase-3 expression after 24 h and 72 h (panel (**A**,**C**)), and on full-length caspase-9 expression after 24 h and 72 h (panel (**B**,**D**)) on PC12 cells. Data are reported as the means ± SD of at least 3 samples for each condition.* Represents a *p* < 0.05, and ** *p* < 0.001 versus control PC12 cells.° Represents a *p* < 0.05 versus G100 mM

Figure 5 shows that glucose affected caspase-3 expression in PC12 cells in a dose-dependent manner (100 and 150 mM) after 24 and 72 h of incubation. At both time points, caspase-3 levels were significantly decreased by the highest glucose concentration (150 mM), with reductions of 2.8-fold and 4.2-fold at 24 and 72 h, respectively. In contrast, treatment with 100 mM glucose did not significantly affect caspase-3 levels (Panels A and C). Regarding caspase-9, glucose at both concentrations (100 and 150 mM) induced a significant decrease in its levels after 24 h of exposure, with reductions of 3.4-fold and 2.8-fold, respectively (Panel B). Conversely, caspase-9 levels were not significantly affected by 100 mM glucose but were significantly reduced by the 150 mM concentration at 72 h, showing a 5-fold decrease compared with both the control and the 100 mM condition (Panel D).

### 3.6. Effects of Glucose Concentrations on PC12 Cells Migration

**Figure 6:**
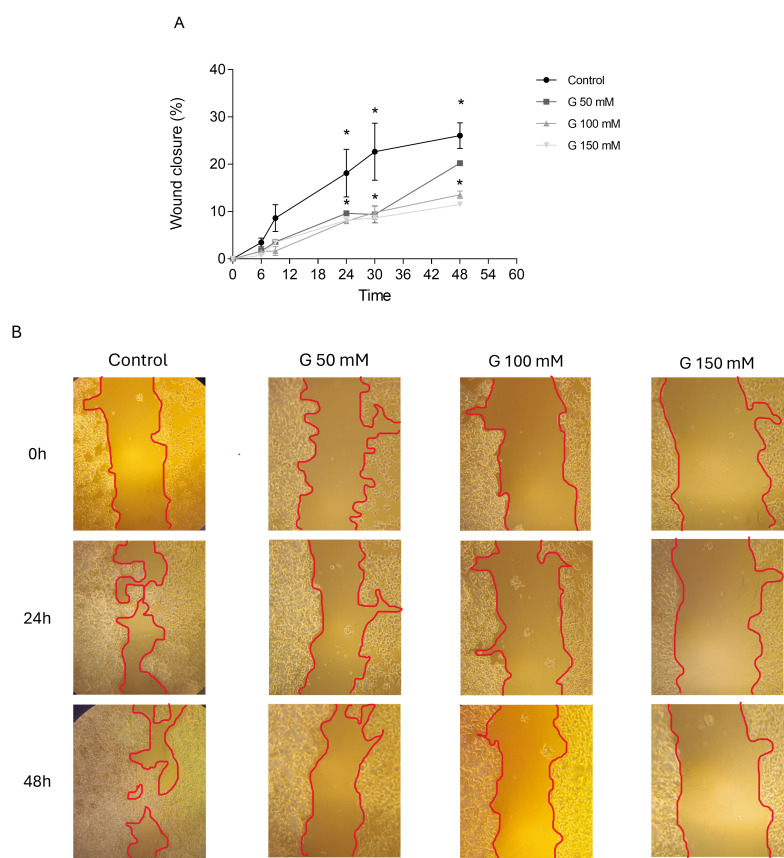
(**A**) Linear regression analysis of data illustrated in (**B**) of migration in PC12 cells pre-treated with glucose at three different concentrations; data are reported as the means ± SD of at least 3 samples for each condition. (**B**) The effect of three different glucose concentrations (50, 100, 150 mM) on the migration of PC12 cells. * *p* < 0.05 vs. control.

Glucose at high concentrations (100 and 150 mM) exhibited significant inhibitory effects on PC12 cell migration from 24 h until 48 h, in comparison with the control group, demonstrating that glucose could slow down the healing of a wound. Indeed, wound closure rates decrease as glucose concentrations increase, as shown by the graph in the Figure 6A,B at 24 h and 48 h (24 h: control 18%, G 50 mM 9%, 100 and 150 mM 8%; 48 h: control 26%, G 50 mM 20%, G 100 mM 13%, 150 mM 11%). Collectively, these results suggest that the tested concentration of glucose (100 and 150 mM) counteracted PC12 cell migration.

## 4. Discussion

Glucose is a molecule known to induce diabetic condition if present at high levels in blood. Hyperglycemia, after persistent episodes in diabetes, which is a chronic metabolic condition, has been shown to lead to a fourfold increase in neuronal glucose levels [17]. The resultant intracellular glucose metabolism can cause neuronal damage, a phenomenon often referred to as glucose neurotoxicity [17]. Therefore, according to Chavda et al., high neuronal glucose levels have been shown to be neurotoxic, foster neurodegeneration, and lead to neuronal dysfunction, a progressive decline in neuronal networks, and cognitive impairment [6]. In addition, it was found that alterations in the brain can modify neurotransmission, which can lead to neuronal loss, demyelination, cognitive dysfunction, vascular dementia, Alzheimer’s disease, and depression in both experimental models and patients [16,26].

These electrophysiological and structural changes, as well as impaired cognitive functioning, are the result of hyperglycemia affecting the CNS [27] and are referred to as “diabetic encephalopathy” [6].

The findings of the present study demonstrate several effects of glucose at different concentrations and during different time points on PC12 cells. The results obtained in this study show that glucose-induced cellular damage in a neuronal model. These effects were achieved by a convergent and complementary approach, through an increase in intracellular production of O_2_^−^, an increase in extracellular levels of MDA, and apoptosis by the reduction in procaspases 3 and 9 and the slowdown in the migration of the cells. Sharifi et al. [20,21] state that high glucose concentrations can significantly decrease the viability of PC12 cells in a time-dependent manner. Several works have reported similar results. For example, Firouzi et al. [28] and Sarvestani et al. [29] considered high glucose-treated PC12 cells to be a model of diabetic neuropathy because of the observed low cell viability and high levels of ROS, MDA, and TNF-α. In addition, Albert-Garay et al. [30] evaluated the effect of high glucose concentrations in rat Müller retinal cells. The authors reported that high glucose-treated cells showed an increase in ROS production but an absence of difference in LDH release between the controls and the treated cells. It is well known that high glucose concentrations induce oxidative stress, which is also involved in the induction of apoptosis. Several reports have described that the overproduction of oxygen radicals, such as O_2_^−^, leads to apoptosis. Li et al. [31] reported that O_2_^−^ is the key precursor of the other ROS generated in mitochondria contributing to oxidative stress, one of the molecular mechanisms involved in neurodegeneration and in the etiology of many diseases, including neurodegenerative disease [32]. Many studies reported the role of O_2_^−^ and oxidative stress in Parkinsons’s disease. For instance, Gonzàlez-Polo et al. [33] reported that some of the most common neurotoxins, useful in inducing parkinsonism in neuronal models, including inhibitors of mitochondrial complex I of respiratory chain, rotenone, or 1-methyl-4-phenylpyridinium (MPP+), are known to increase oxidative stress and produce superoxide anions in submitochondrial particles. Mitochondrial complex I, in the event of mutation, has been demonstrated to result in neurodegeneration. As Abramov et al. [34,35] demonstrate in their study, mutations in mtDNA in neurons derived from stem cell cybrids induce cell death by overproduction of superoxide in the matrix of mitochondria. The previously mentioned research support the results obtained in our study, where glucose at the highest concentrations (100 and 150 mM) induced significant and dose- and time-dependent increases in O_2_ but did not induce any changes in ROS levels between controls and treated cells. In accordance with our result, Graier WF et al. [36] also demonstrated that high glucose concentrations increase O_2_^−^ levels in endothelial cells. The authors hypothesize that the glucose-induced increase in O_2_^−^ results in alterations in endothelial Ca^2+/^EDRF signaling, leading to an augmentation in Ca^2+^ levels, an increase in EDRF (endothelium-derived relaxing factor), and consequently to vasodilatation [36].

In addition, our results confirmed oxidative damage, suggesting that the highest concentrations of glucose (100 and 150 mM) induced an increase in lipid peroxidation (MDA) in PC12 cells. Thakkar et al. [37] demonstrated that MDA is able to induce modifications in α-synuclein, which consist of alterations of its structural properties and enhancement of aggregation propensities. These modifications could contribute to neurodegeneration, a decline in motor functions and aggregation of proteins in the brain, as observed in Parkinson’s disease (PD) models. In addition, Kaeidi et al. [38] showed, with their investigation, the increase in oxidative stress by excessive MDA generation, in PC12 cells treated with high glucose (100 mM).

Sidorova et al. [39], in their review reported, in the context of oxidative stress, that elevated concentrations of MDA have been testified in the plasma and cerebrospinal fluid of PD patients and that lipid peroxidation products are able to modify α-synuclein and consequently disturb the mitochondrial function.

In this study, we also evaluated nitrite levels in PC12 cells and found no significant differences between the control group and glucose-treated cells, except for the experimental condition of glucose 100 mM at 24 h. Our result may be explained by the findings of Vassalle et al. [40], who reported that NO levels may be inversely proportional to MDA levels. In their study, the authors assessed plasma levels of nitrite plus nitrate (NOx) and MDA as indicators of NO release and lipid peroxidation, respectively, in sedentary individuals, athletes, and heavy smokers. They suggested that a sequence of factors leading to reduce NO production or increased NO degradation due to oxidative stress can result in elevated MDA levels and enhanced lipid peroxidation.

In addition, it has been reported in the literature that several factors may affect the balance of NO and O_2_^−^ under high-glucose conditions. Noyman et al. [41] state that the imbalance in the decreased production and activity of NO, or in the incremented production of free radicals, such as O_2_^−^, may induce a compromising of endothelial relaxation in blood vessels. This hypothesis could explain the results obtained in our study, in which we demonstrated an increase in O_2_^−^ levels, but a reduction in nitrite levels in some reported experimental conditions. Therefore, in accordance with the preceding discourse, the findings of this study are consistent with the established principle that glucose, through its capacity to elevate MDA and O_2_^−^ levels, consequently, incites an escalation in apoptosis. These activities could be the primary mechanisms underlying the cytotoxic effect of glucose. Indeed, under the experimental conditions used, treatment with glucose at the highest concentrations (100 and 150 mM) significantly reduced the expression of full-length caspase-3 and caspase-9 compared with control conditions, suggesting a possible increase in the cleaved and active forms of these proteins [42]. The decrease in the expression of full-length caspase-3 and caspase-9 has been confirmed by Sharifi et al. [21], which suggested that caspases are activated following high glucose treatment and are needed to induce apoptosis in PC12 cells. The results of their study reported that high glucose increased the enzyme activity of these caspases, which was paralleled by a decrease in expression of pro-caspase-8, -9, and -3 at 72 h post-treatment. Additionally, it is known that hyperglycemia is a critical mechanism in the induction of oxidative stress. Oxidative stress is known to control the complex process of diabetic wounds’ healing through several related signal pathways. This detail could be confirmed by the fact that antioxidants and antioxidative enzyme systems may reduce oxidative stress-induced damage to improve wound healing [43].

Our results have highlighted that glucose, at high concentrations, induces a significant slowdown of PC12 cell migration and wound closure. These data show that glucose at the concentrations used plays an important role in wound healing in several cell models. For instance, as demonstrated by Buranasin et al. [44] in human gingival fibroblasts, glucose at elevated concentrations has been shown to significantly retard migration in comparison with the control group. In their study, Zhu et al. [45] investigated the impact of elevated glucose levels on the morphology of primary hippocampal neurons and the microfilament cytoskeleton. The results of the study demonstrated that elevated glucose levels resulted in a reduction in synapse size, nuclear shape irregularities, and microfilament damage. The rearrangement of filamentous actin (F-actin) filaments was observed, resulting in an increase in the rate of cell apoptosis. Furthermore, an increase in the protein expression of myosin light chain kinase (MLCK) and phosphorylated MLC (pMLC) was also noted. In summary, the authors indicated that elevated glucose levels resulted in the upregulation of MLCK, thereby promoting the depolymerization of F-actin. This, in turn, induced a rearrangement of the microfilament cytoskeleton in neuronal cells of the hippocampus. Consequently, we considered the eventual hypothesis that elevated glucose concentrations may induce alterations in cellular morphology and cytoskeleton in our neuronal model and then could influence its migratory ability.

## 5. Conclusions

This study evidenced neuronal toxicity from high glucose concentrations in PC12 cells, through a reduction in cell viability, an increase in superoxide anion and MDA levels, a reduction in caspase-3 and -9 precursors, and a slowdown in cell migration. This work could be a starting point to study the high glucose-induced injury and its multifactorial impact on several neuronal models, as well as for the investigation of the relationship between high glucose concentrations and neurodegeneration. In our work, PC12 cells were used as a valuable model for studying the mechanisms underlying glucose-induced damage, although further research is needed to fully elucidate the molecular pathways involved. Although confirmatory in nature, the study adds valuable molecular insights that contribute to a better understanding of the fundamental mechanisms underlying neuronal damage. Furthermore, it offers useful perspectives for expanding and validating these findings in different neuronal models.

## Data Availability

Data will be made available on request.
